# Mapping Multi-Disease Risk during El Niño: An Ecosyndemic Approach

**DOI:** 10.3390/ijerph15122639

**Published:** 2018-11-25

**Authors:** Ivan J. Ramírez, Jieun Lee, Sue C. Grady

**Affiliations:** 1 Department of Geography and Environmental Sciences, University of Colorado Denver, Denver, CO 80217-3364, USA; 2 Consortium for Capacity Building/Institute for Arctic and Alpine Research, University of Colorado Boulder, Boulder, CO 80309-0450, USA; 3 Department of Geography and GIS, University of Northern Colorado, Greeley, CO 80639, USA; Jieun.Lee@unco.edu; 4 Department of Geography, Environment, and Spatial Sciences, Michigan State University, East Lansing, MI 48824, USA; gradys@msu.edu

**Keywords:** syndemic, El Niño, infectious disease, diarrhea, malaria, respiratory, cholera, spatial cluster, GIS

## Abstract

El Niño is a quasi-periodic pattern of climate variability and extremes often associated with hazards and disease. While El Niño links to individual diseases have been examined, less is known about the cluster of multi-disease risk referred to as an ecosyndemic, which emerges during extreme events. The objective of this study was to explore a mapping approach to represent the spatial distribution of ecosyndemics in Piura, Peru at the district-level during the first few months of 1998. Using geographic information systems and multivariate analysis, descriptive and analytical methodologies were employed to map disease overlap of 7 climate-sensitive diseases and construct an ecosyndemic index, which was then mapped and applied to another El Niño period as proof of concept. The main findings showed that many districts across Piura faced multi-disease risk over several weeks in the austral summer of 1998. The distribution of ecosyndemics were spatially clustered in western Piura among 11 districts. Furthermore, the ecosydemic index in 1998 when compared to 1983 showed a strong positive correlation, demonstrating the potential utility of the index. The study supports PAHO efforts to develop multi-disease based and interprogrammatic approaches to control and prevention, particularly for climate and poverty-related infections in Latin America and the Caribbean.

## 1. Introduction

The aim of this study is to explore mapping multi-disease risk during El Niño-Southern Oscillation (ENSO) and hydro-meteorological extremes in northern Peru. It builds on previous climate-health research in South America [1,2] and expands current work on El Niño-related disasters [3,4,5]. ENSO is a climate variability pattern that affects local to global weather patterns every 2 to 8 years. It stems from oceanic-atmosphere interactions in the equatorial Pacific Ocean and has three phases that consist of El Niño (warm extreme), La Niña (cold extreme), and Neutral conditions [6]). The El Niño phase is often associated with hydrometeorological hazards (e.g., temperature extremes, floods, and windstorms) with untoward effects on societies, including infectious disease outbreaks [7]. 

In northwest South America, during El Niños, the region experiences the emergence and resurgence of water-borne and water-based infectious diseases, such as, cholera (e.g., [8]), malaria (e.g., [9]), dengue (e.g., [10]), and most recently, Zika (e.g., [11]). El Niño impacts disease ecology, both directly and indirectly, by altering temperature and rainfall patterns, as well as inducing sea level and marine ecosystem changes that affect water-related pathogens [1]. For example, elevated air and water temperatures can lead to the multiplication of pathogens in food and water (e.g, *vibrio cholerae*, the causative agent of cholera) and heavy rains can transport pathogens along waterways [12,13], increasing the likelihood for human disease exposures. Furthermore, flooding along with coastal storm surge impacts urban built environments, which can lead to the breakdown of infrastructure systems, including water and sanitation [14], which in turn affect a myriad of gastrointestinal, respiratory, rodent-borne, and ocular infections [15,16]. Hydrometeorological extremes also affect a variety of disease vectors, including mosquitoes, which transmit malaria caused by a protozoan parasite [17]. For example, local temperature can directly affect the survival and reproduction of the vector and parasite, as well as vector activity [18,19]. Many of the aforesaid infections are also linked to pressures from urbanization and social inequality [20,21] that influence population vulnerability to old and new agents and vectors of disease [22,23,24].

While many studies have focused on El Niño’s link to one infectious disease at a time, less is known about El Nino’s impact(s) on the clustering of several infectious disease outcomes in a population also known as an ecosyndemic [25]. The concept of ecosyndemic, which builds on the notion of syndemic [26], describes the overlap of infectious diseases spawned by climate and environmental changes in vulnerable places. It frames disease from a view of a broader health problem (i.e., multiple diseases potentially interlinked) that highlights climate processes but also emphasizes the role of human activities (e.g., urbanization) and social determinants of health (i.e., the inequality of social conditions in which people live). Ecosyndemics are important to consider because of their potential interactive and cascading effects on public health. For example, Singer [27] suggests the rise in asthma is a consequence of an increasing hazardous environment, where respiratory health risks interact (e.g., asthma, allergic rhinitis and viruses), induced by air pollution, exacerbated by global warming, and facilitated by “social relationships.” While a conceptual description of what an ecosyndemic has been discussed, the spatial patterns of such public health phenomena have not been explored. Thus, this present work seeks to address this gap by exploring an ecosyndemic approach to mapping multi-infectious disease risk using Piura, a northwest region in Peru, as a case study during the well documented El Niño in 1998. 

In 1998, like in 1983, multiple infectious disease epidemics were reported in Piura and Peru in general following torrential rains and flooding associated with one of the strongest El Niños of the 20th century [28,29,30]. Many of these diseases were already endemic problems such as acute diarrheal diseases, respiratory-related infections, and two types of malaria, which increased substantially from previous years in 1982 and 1997 [1,28,30]. More recently, El Niño was linked to increased incidence of arboviruses in Piura in 2017 [31]. Thus, in the time of El Niños, the health sector in Piura responds to an excess burden of disease and health afflictions. 

To examine multi-infectious disease risk in Piura, this study used geographic information systems (GIS) to characterize and visualize the geography of ecosyndemics at the district-level, including the detection of significant clustering areas in the austral summer of 1998. GIS is an important public health tool by which to model and explore the spatial patterns of disease, risk factors and clustering [32,33]. Specifically, two methodologies were employed to map disease overlap and construct an ecosyndemic index, which was then mapped and applied to another El Niño period as proof of concept. Such an approach and mapping tools can better enable the public health sector to identify areas of multi-infectious disease risk. Potentially this effort can support multi-infectious disease-based programs for surveillance, control, and prevention suggested by the Pan American Health Organization (PAHO) for the broader Latin American and Caribbean region [34]. 

## 2. Materials and Methods 

### 2.1. Mapping Approach

In this study, we explored two methodologies to represent and map ecosyndemics inspired by PAHO initiatives for vector-borne and neglected tropical diseases [34,35,36]. The aims of these programs are to reduce and eliminate the disease burden of preventable infections in vulnerable places using integrated and comprehensive approaches, including descriptive risk maps. The motivation is to identify an overlap of diseases related to poverty and environment so that “We can spread…benefits to the community with the same intervention” [34]. 

One way to represent ecosyndemic is based on the work of Schneider et al. [36]. Using various geoprocessing tools, the authors provided a practical methodology for mapping the overlap of several neglected tropical diseases at the subnational-level in Latin America and the Caribbean (LAC). For example, GIS maps of prevalence for individual diseases (‘hotspots’) were visualized and overlapping disease areas (‘major hotspots’), which were classified by 0 to 4 diseases present, were identified. Diseases transmitted by climate sensitive vectors—fleas and flies including lymphatic filariasis, onchocerciasis, and trachoma were selected based on potential for elimination and drastic reduction, as well as data availability. 

Another way to represent ecosyndemic is based on a composite index, which can then be mapped. Composite indices are often used for characterizing the well-being and vulnerability of populations and places, such as the Human Development Index [37], social vulnerability index (SVI) [38], or a basic needs index [39]. Typically, they are static measures (snapshots in time) composed of several quantitative indicators for socioeconomic status, education, infrastructure, shelter, livelihoods, as well as health. A conceptual framework often guides the index construction process including what the index intends to characterize and represent [40,41,42]. Variables for the index are selected based on relevance to the issue or problem, review of the literature, statistical analysis, and availability of data. Selection is also influenced by expert input or a combination of factors [40,42]. Geographical scale is important to index construction because the level of analysis can influence the association derived, and may vary by scale [41], (e.g., different outcomes when comparing national and subnational levels). Most commonly the aggregation of variable inputs to the index are achieved through an additive model, where indicators are summed, then divided by the number of indicators (e.g., the SVI) [41]. Aggregation is also achieved through multivariate methods such as principal components analysis (PCA), where data structure and interactions are accounted for and orthogonal components are the output [42]. Using those components, the additive model is then used to calculate the composite index. 

Disease-related indices are less common, but one example of a composite disease index is presented by Confalonieri et al. [43]. The authors developed an epidemiological indicator within a public health vulnerability index for Brazil. This indicator based on seven endemic diseases (sub-indicators), including cholera and malaria, represented population sensitivity to climate change. The epidemiological indicator was composed of incidence rates, hospital admissions, mortality rates, and hospital admissions costs. Each disease was of public health importance in Brazil and represented one dimension of epidemiological vulnerability, which was calculated using an additive model (based on HDI) and scaled from 0 to 1. Weights were added to each disease based on expert input according to several characteristics, including knowledge on drug resistance and fatality rates. The final indicator was then mapped from highest to lowest vulnerability at the state-level to demonstrate the utility of such an index in identifying where to intervene. 

While Confalonieri et al. [43] offers a potential approach to ecosyndemic index construction, our study did not have similar datasets and resources available such as the local expert input to apply such a method. Furthermore, our disease variables were correlated to some extent and Confalonieri et al. [43] did not address this statistical issue. For the purpose of our study, we used the composite approach developed by Cutter et al. [38] for the SVI, mentioned earlier, to construct an ecosyndemic index. This method was chosen because it provides simple steps to synthesize the ensemble of diseases utilized in this study and considers collinearity among variables. Conceptually for our analysis, we defined ecosyndemics as infectious disease hotspots in places (i.e., districts) where two or more diseases are concentrated, which can potentially afflict a population during and following climate extremes. For the index, seven diseases were selected based on a set of infections that commonly spike during and after El Niños identified by the regional public health authorities in Piura [29]. The temporal resolutions are week, month, and season, and the geographic level of analysis is the district—the finest scale available for the disease data and better represents disease concentration in time at a local level. 

In this study, we adopted two methodologies to explore an ecosyndemic approach to mapping multi-disease risk. The first is a descriptive method influenced by PAHO to classify and map the number of diseases present. It provides a crude and practical measure of ecosyndemics for practitioners. The second is an analytical method that uses a composite index to aggregate several diseases and account for their interactions. It offers a more complex representation of ecosyndemic. The details of these methodologies are described in the following sections below. 

### 2.2. Study Area

**Figure 1:**
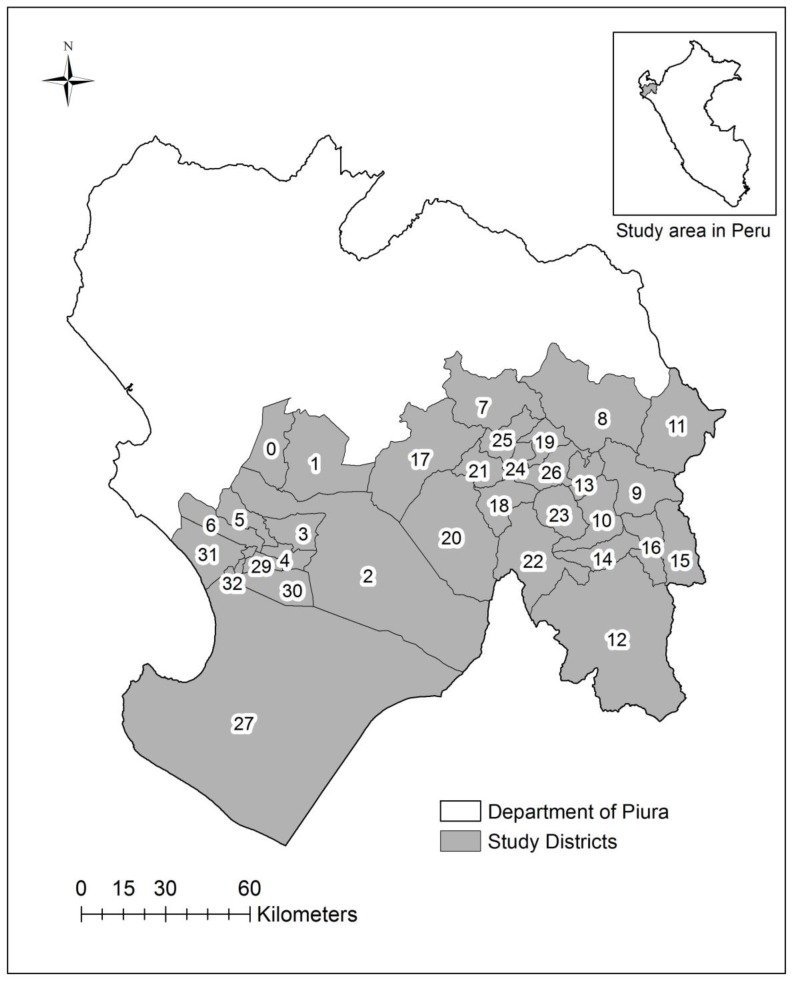
Map of the study area including the districts in Piura, Peru (n = 33): Piura (0), Castilla (1), Catacaos (2), Cura Mori (3), El Tallan (4), La Arena (5), La Union (6), Frias (7), Pacaipampa (8), Huancabamba (9), Canchaque (10), El Carmen de la Frontera (11), Huarmaca (12), Lalaquiz (13), San Miguel de el Faique (14), Sondor (15), Sondorillo (16), Chulucanas (17), Buenos Aires (18), Chalaco (19), La Matanza (20), Morropon (21), Salitral (22), San Juan de Bigote (23), Santa Catalina de Mossa (24), Santo Domingo (25), Yamango (26), Sechura (27), Bellavista de la Union (28), Bernal (29), Cristos Nos Valga (30), Vice (31), Rinconada Llicuar (32).

Piura is a coastal area located approximately 1000 km north of Lima, Peru (Figure 1). It is a place well known for El Niño impacts [44], adjacent to the Pacific Ocean which stretches to the foothills of the Andes. Its regional climate varies from semi-arid to tropical dry mountain savanna. Administratively, it is one of two health subregions in the Department of Piura, which is the second most populous state in Peru. The subregion Piura is divided into 33 districts. The total population in 1998 was 847,257 [45]. 

### 2.3. Data Processing

#### 2.3.1. Climate Data 

Sea surface temperature (SST, °C) variables in the equatorial Pacific Ocean, such as Niño 3.4 SST (5° N–5° S and 170°–120° W), Niño 3 (5° N–5° S and 90°–150° W), and Niño 1 + 2 (0–10° S and 80°–90° W) at a weekly scale from 1981 to 2000 were obtained from the Center for Climate Prediction [46]. Timeplots of these climate data provide descriptive information of the El Niño context preceding and during the study period.

#### 2.3.2. Epidemiological Data

Infectious disease data were obtained at the weekly level and aggregated to monthly and seasonal time frames using the suspected and probable case reports from January to March 1998 [29]: acute diarrheal diseases excluding cholera (EDAS), cholera (CHOL), acute respiratory infections excluding pneumonia (IRAS), pneumonia (PNEU), conjunctivitis (CONJ), and two types of malaria *P. falciparum* (MALFP) and *P. vivax* (MALV). From these data, disease percentages and incidence rates at weekly, monthly, and seasonal scales (aggregated January, February, and March) were calculated. The disease percentage describes the relative contribution of each disease to the overall disease incidence per week (all seven diseases in this study). Percentages for each disease were estimated by dividing the number of cases of each disease by the total number of cases for all seven diseases multiplied by 10^2^ (per 100 persons). Disease incidence rates were calculated by dividing the total number of cases of each disease by population at risk estimates in 1998 and multiplying by 10^3^ (per 1000 persons) for each district and subregion (all districts). In addition, disease data associated with El Niño impacts in 1983 were obtained from Peru’s census bureau, INEI [47]. These data consisted of suspected and probable cases including some of the infections reported in 1998 (e.g., EDAS, IRAS, and malaria). These data were only available at the seasonal scale and were utilized for comparative purposes to demonstrate a proof of concept (application of methodology to 1983). The surveillance definition of suspected cases were observations of signs and symptoms documented by a physician or other health care provider. 

#### 2.3.3. Social and Disaster Data

Social data at the district-level consisting of a basic needs index (percentage of population with at least one basic need unmet) and select individual basic needs variables, such as percentage of population without municipal water or electricity in their households were used as a proxy for population vulnerability in Piura, Peru. The basic needs index and census data were available for 1993 [48], which is the most relevant census assessment that precedes the study time period. In addition, damages data associated with the 1997–1998 El Niño such as the number of people affected at the district-level from January to May 1998 were obtained from INEI [49]. 

### 2.4. Characterizing and Mapping Ecosyndemics

#### 2.4.1. Mapping Overlapping Diseases

The overlap of infectious diseases (*n* = 7) was measured by identifying the presence of each disease by place (district) and time (week) across 12 weeks. The presence of a disease was defined as at least one case reported per week. At least one case of disease is used to document the presence of disease in that district and to account for other potential undocumented cases in the place. 

#### 2.4.2. Mapping an Ecosyndemic Index 

Using a method from Cutter et al. [38], a composite index was constructed to estimate ecosyndemics for each time scale: weeks (1–12), months (January, February and March), and season (January, February and March). The ecosyndemic index was generated using PCA in IBM SPSS (IBM Corp, Armonk, NY, USA). Prior to analysis, all disease variables were standardized by calculating z-scores (standard deviations) and assessed for collinearity using Pearson’s correlation. PCA is a method that addresses issues of multicollinearity and creates orthogonal dimensions that can be used in a composite index. Factor scores which represent how influential a disease is in a composite data structure are derived from the PCA, then summed, and divided by the number of dimensions. The composite index was then scaled from 0 to 1 using a method by Confalonieri et al. [43].

#### 2.4.3. Spatial-temporal and Cluster Analysis

Several geoprocessing tools and methods in ArcGIS (ESRI, Redlands, CA, USA) were utilized for spatial-temporal and cluster analysis of individual and composite infectious diseases and ecosyndemic patterns. For each disease, choropleth maps were created to visualize incidence rates at the seasonal scale. In addition, for each disease, timeplots of the weekly incidence for the subregion were processed to describe temporal patterns of disease. To visualize ecosyndemics, index values were transformed into points and then interpolated using an inverse distance weighted (IDW) algorithm to create a continuous surface for mapping. IDW assumes that values closer to a data point have a stronger influence and diminishes with distance [50]. By doing so, crude visualizations of ecosyndemic hotspots were generated for sequential weekly, monthly, and seasonal scales. To characterize spatial patterns of ecosyndemics, Global Moran’s *I* and Hotspots Analysis (Getis-Ord Gi* statistic), common tools for spatial analysis [51], were used to assess (a) spatial dependency relationships (spatial autocorrelation); and (b) to detect where statistically significant spatial clusters of high values (hot spots) and low values (cold spots) occurred. 

#### 2.4.4. Application of Ecosyndemic Index

To test the useability of the ecosyndemic index (proof of concept), Pearson’s correlation analysis was conducted in IBM SPSS to estimate the relationships between El Nino, the basic needs of people and disaster outcomes and the ecosyndemic index. These data and their associations provide a deeper understanding of the spatial patterns of ecosyndemics. Secondly, the index methodology was applied to disease morbidity associated with the 1982–1983 El Niño in the region. For temporal comparison, common disease variables were selected for index construction for the 1983 and 1998 study periods: suspected and probable cases of acute diarrheal diseases, acute respiratory infections, and malaria (*P. vivax*). Furthermore, analysis was restricted to districts, where ecological data were complete for the three disease variables reported previously. Ecosyndemic indices for 1983 and 1998 were then ranked, mapped, and evaluated for correlation for comparison. 

## 3. Results

### 3.1. Climate Context

**Figure 2:**
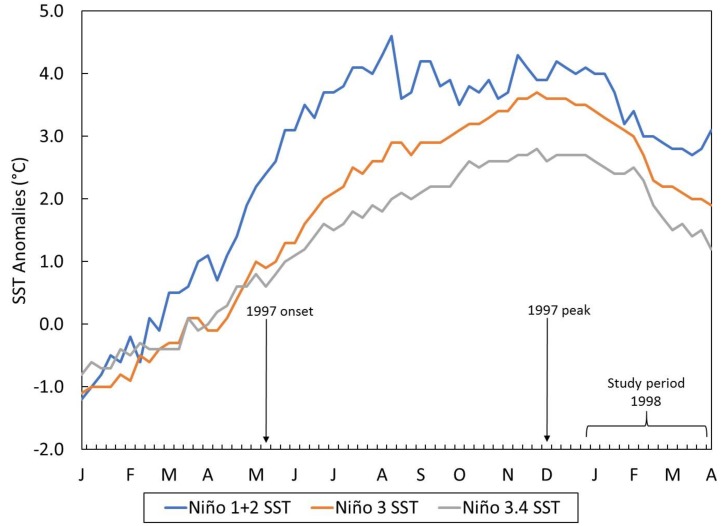
Weekly time plot of sea surface temperature anomalies (°C) in the central eastern (Niño 3.4), eastern (Niño 3) and coastal upwelling (Niño 1 + 2) regions of the equatorial Pacific Ocean from January 1997 to April 1998. El Niño onset and peak (based on ONI index) and study period are highlighted.

The El Niño context associated with the study time period is presented in Figure 2, which shows SST anomalies across several regions in the equatorial Pacific Ocean from January 1997 to April 1998. 

According to the ONI index [46], the development of the mega El Niño began in May 1997, peaked in December, and subsided by May 1998 (data not shown). The most intense SST deviations were reported in the upwelling and coastal regions of northern Peru and southern Ecuador (Niño 1 + 2) followed by high but weaker anomalies as one moved west along the equator. 

### 3.2. Incidence Disease Burden

Following the peak of El Niño, from January to March 1998, there were 80,600 reported cases of infectious diseases relevant to this study and the overall incidence rate was 9.5 per 1000 population in the subregion of Piura, Peru. The relative contribution of each disease to the total disease burden, measured as a percentage, is shown in Table 1. Acute respiratory infections followed by diarrheal-related non-cholera diseases represented a majority of infections (79.1%) for the entire season. The maximum percentages (peaks) of disease by week (WK) were as follows: diarrheal disease non-cholera (EDAS) (WK1: 30.0%), malaria *P. falciparum* (MALFP) and malaria *P. vivax* (MALV) (WK3: 8.2% and 7.7%), acute respiratory infections (IRAS) (WK6: 60.2), cholera (CHOL) (WK8: 7.2%), conjunctivitis (CONJ) (WK10: 10.4%), and pneumonia (PNEU) (WK12: 5.7%). 

### 3.3. Temporal Patterns of Incidence Rates

**Figure 3:**
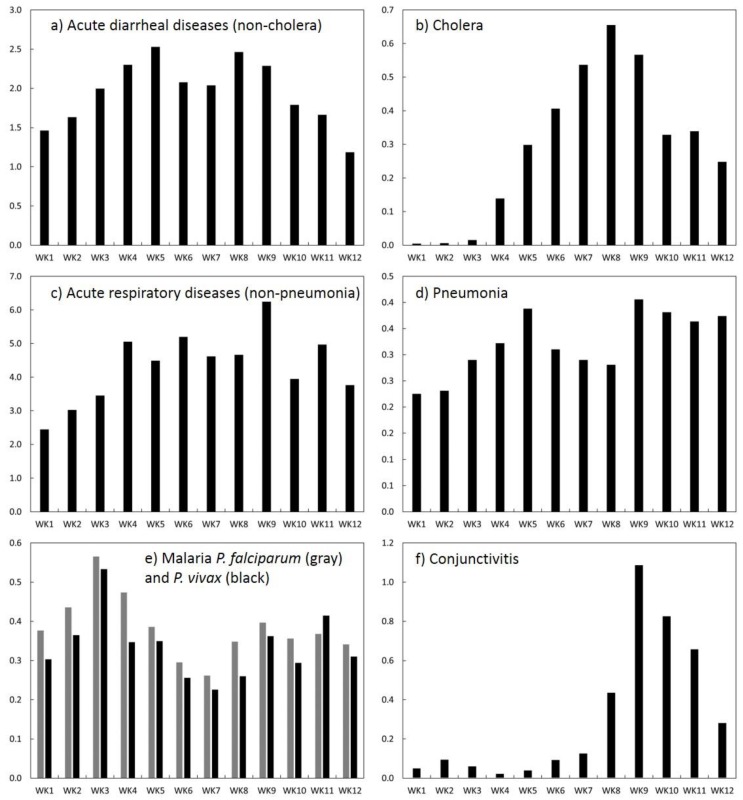
Weekly time plots of disease incidence (per 1000 population) for the study area (all districts): (**a**) EDAS (acute diarrheal diseases—non-cholera); (**b**) cholera; (**c**) IRAS (acute respiratory diseases—non-pneumonia); (**d**) pneumonia; (**e**) malaria P. *falciparum* and malaria P. *vivax*; (**f**) conjunctivitis.

Time plots of the weekly incidence rate (per 1000 population) for each disease are shown in Figure 3 (see Appendix A, Table A1 for weekly incidence rates). Incidence rates of diarrheal diseases—non-cholera (EDAS) and pneumonia (PNEU) increased markedly within the first few weeks of 1998, peaked at week 5 (EDAS: 2.5; PNEU: 0.4), subsided, and then peaked again at week 9 (EDAS: 2.3; PNEU: 0.4), although EDAS sharply declined thereafter. Overall, EDAS remained above 1.4 (per 1000) throughout most of the study period suggestive of sustained water and/or food-borne transmission. The incidence rate of IRAS was the highest among diseases, remained above 4.0 (per 1000) from weeks 4 to 9 (peak at 6.2 per 1000) suggestive of increasing susceptibility resulting in upper respiratory infections. Incidence of both malarias followed a similar temporal pattern. Each disease peaked in the 3rd week (~0.5–0.6), subsided through weeks 6 and 7, and then increased again by week 9 (0.4). Conjunctivitis (CONJ) and cholera (CHOL) rates, on the other hand, peaked later in the season; they climbed markedly by weeks 8 (CHOL: 0.7) and 9 (CONJ: 1.1) suggestive of heightened susceptibility and increasing exposures due to extreme weather and water contamination. In the case of cholera, disease incidence resurged during this time period after a decline in the past year [1]. Overall, the total incidence rate had a minimum value at week 1 (.5 per 1000) and peaked at week 9 (1.1 per 1000). 

### 3.4. Spatial Patterns of Incidence Rates

**Figure 4:**
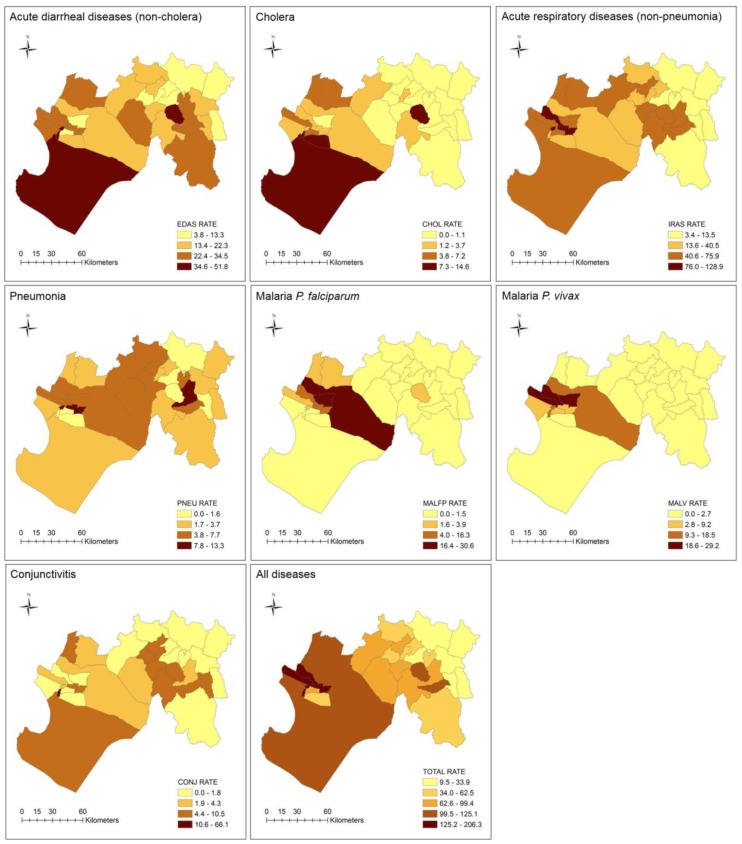
Maps of disease incidence rates (per 1000) at the district-level for the entire season, natural breaks classification: EDAS (acute diarrheal diseases—non-cholera); cholera; IRAS (acute respiratory diseases—non-pneumonia); pneumonia; malaria P. *falciparum*; malaria P. *vivax*; conjunctivitis; and total (all diseases).

The spatial patterns of incidence rates (per 1000 population) are visualized at a seasonal scale in Figure 4 and Table 2. The highest rates for EDAS and IRAS were distributed across the subregion, although for IRAS, there were more western districts with greater incidence. High rates of CONJ were generally found in western and eastern districts, whereas PNEU rates were concentrated in the center with a few high-rate districts in the east. Incidence of CHOL and both malarias have distinct patterns concentrated in the west of the subregion, except for one eastern district which stands out in terms of cholera incidence (i.e., San Juan de Bigote: 10.6 per 1000). Overall, the total incidence rates (all diseases) were mainly found in western districts, except for the eastern districts of San Juan de Bigote (121.0 per 1000), described earlier, and San Miguel de el Faique (110.0 per 1000).

### 3.5. Incidence Burden Ratio by District

Table 3 shows the disease burden ratios (i.e., number of times higher for each disease compared to the average) by district for the season. Some of the highest rates based on the burden ratio were reported in Rinconada Llicuar (CONJ: 12), Catacaos (MALFP: 9), Cura Mori (MALFP: 8; MALV: 5), Cristos Nos Valga (CHOL: 5), La Arena (MALFP: 5; MALV: 6), La Union (MALV: 5), Bellavista de la Union (MALV: 4), El Tallan (MALFP: 4), and San Juan de Bigote (CHOL: 4). Based on the total incidence rate, the districts with the greatest burden ratio (2 times higher) were Bellavista de la Union, El Tallan, La Arena, La Union, and Rinconada Llicuar. The districts with the lowest burden ratio (less than 1) were Chalaco, El Carmen de la Frontera, Huancabamba, Huarmaca, Pacaipampa, Sondor, Sondorillo, and Yamango. 

### 3.6. Mapping Multi-Disease Risk

#### 3.6.1. Spatial Patterns of Overlapping Diseases

**Figure 5:**
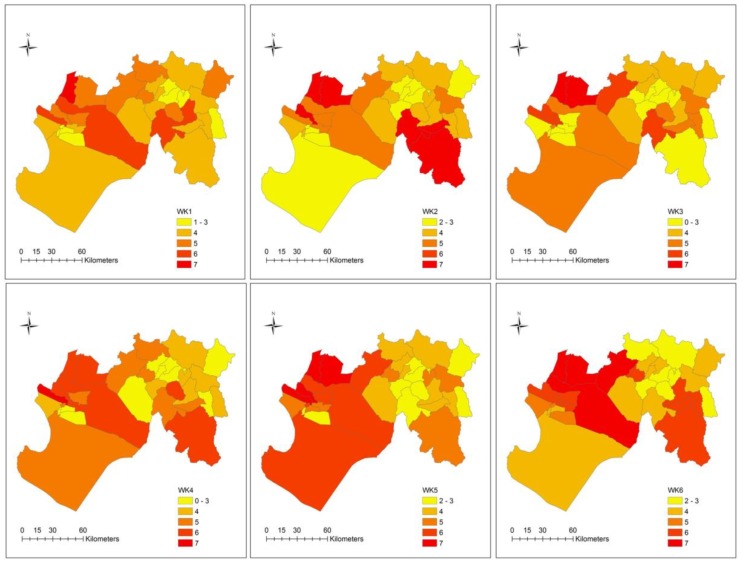

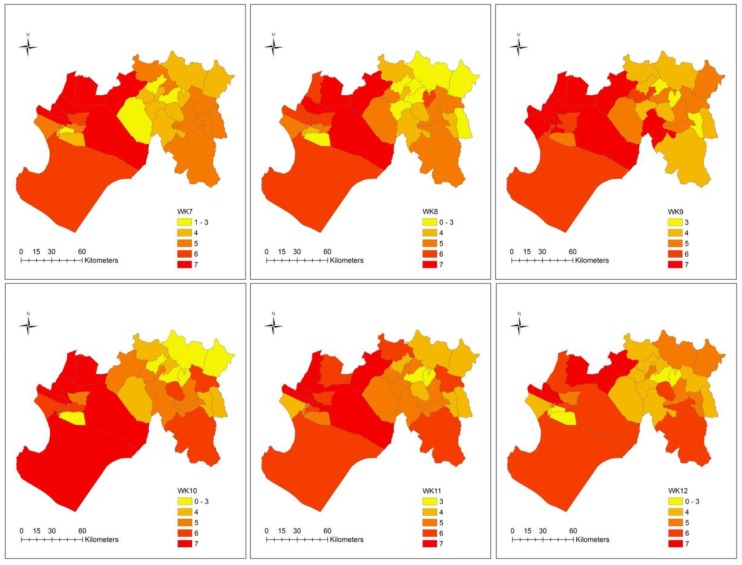
Maps of the number of overlapping diseases at the district-level by week, manual classification.

The spatial distribution of overlapping diseases for all 12 weeks is displayed in Figure 5. In general, many districts reported 2 or more diseases per week. Several districts reported 6 to 7 diseases, a pattern which appeared to intensify (i.e., as the number of overlapping disease increased) and concentrate in the west, particularly after weeks 5 and 6. As an example, during week 9 approximately 70.0% of districts (*n* = 23) reported at least 5 or more diseases (see Appendix A, Table A2). Even the eastern district of Yamango, which exhibited a low disease incidence burden (see Table 2), reported at least 5 diseases in week 9. Of those high burden districts in week 9, 39.1% reported all 7 diseases. Overall, districts in the east appeared to be less vulnerable to disease overlap, although the southeast district of Huarmaca stands out in weeks 2 and 6. 

#### 3.6.2. Ecosyndemic Index

An ecosyndemic index, which represents the overlap of disease morbidity by district, was generated for each week, month, and season from January to March 1998. Prior to data analysis, correlations between the disease variables were assessed (see Appendix A, Table A3). The strongest positive relationships were between the two types of malaria (*r* = 0.7, *p*-value < 0.01). There were also moderate positive correlations between IRAS and PNEU (*r* = 0.6, *p*-value < 0.01), EDAS (*r* = 0.5, *p*-value < 0.01), and MALV (*r* = 0.5, *p*-value < 0.01). EDAS were positively associated with CONJ (*r* = 0.5, *p*-value < 0.01) and CHOL (*r* = 0.5, *p*-value < 0.01). Using PCA, multicollinearity among the disease variables was addressed and a composite index was generated. For the sake of brevity, the PCA output, including the principal components (Comp.), percentage of variance (Var.) and cumulative percentage of variance (Cumul.) accounted for by the current and all preceding components, from the monthly index is described here in Table 4 (see Appendix A, Table A4 for the PCA output for the weekly and seasonal indices). 

Factor loadings above 0.60 are highlighted (bold) and represent dominant variables in the components (also known as dimensions) derived from the PCA. According to the analysis, the IRAS and PNEU factors are consistently found within the first component, which explained most of the variance in the data (above 34.08%). For all other variables, which factors are dominant by component varied from month to month. The cumulative percentage of variance explained most was found in the February index (3 components which explained 80.07% of the variance).

#### 3.6.3. Spatial Patterns of the Ecosyndemic Index

**Figure 6:**
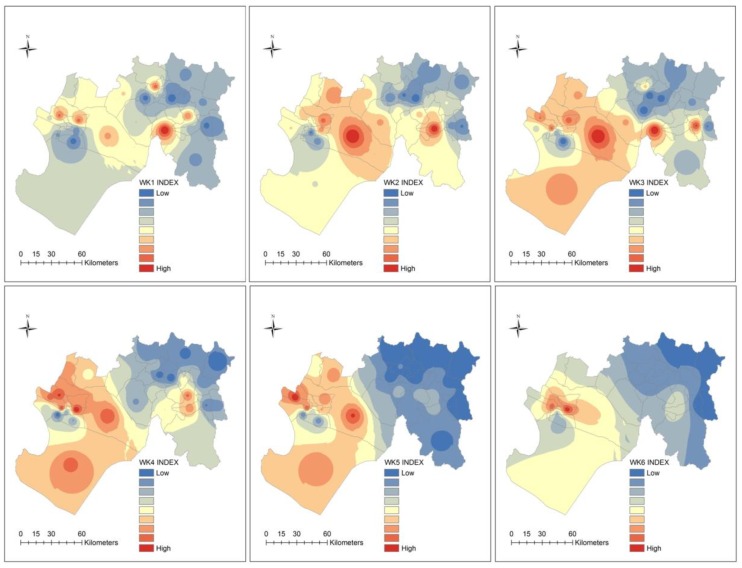

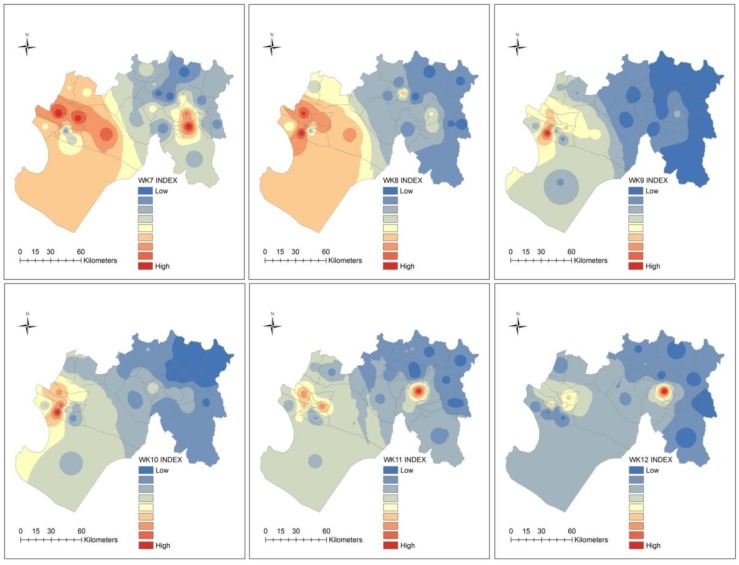
Maps of the ecosyndemic index (low—blue to high—red) at the district-level by week, using the inverse-weight distance interpolation method.

**Figure 7:**
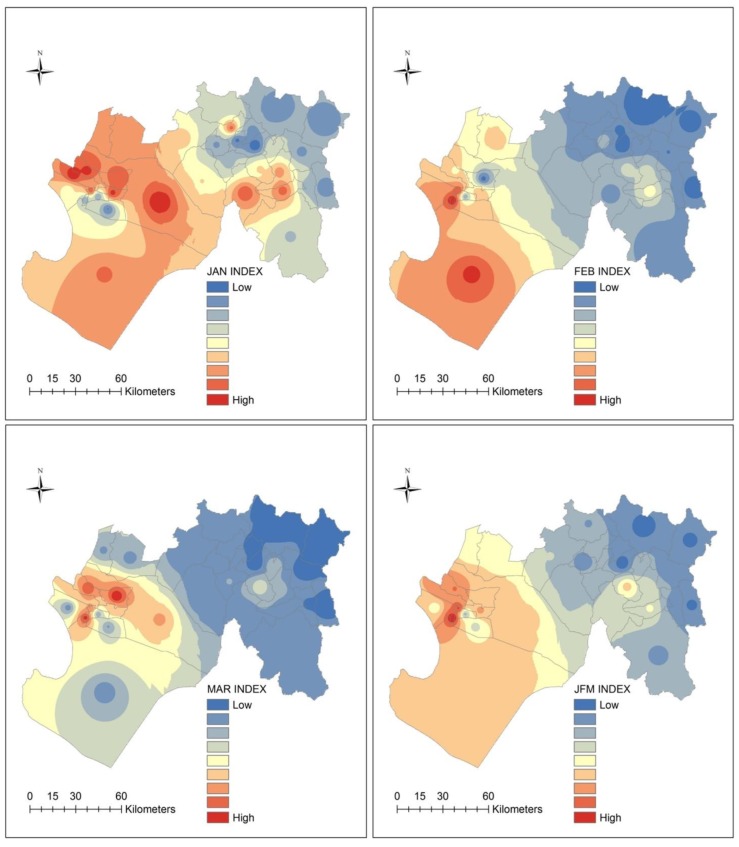
Maps of ecosyndemic index (low—blue to high—red) at the district-level by month and season (JFM), using the inverse-weight distance interpolation method.

Maps of the ecosyndemic index by week, month and season are shown in Figure 6 and Figure 7. The ecosyndemic pattern indicates that multi-disease morbidity (red values represent crude hotspots of risk) initially spreads from the center of the subregion and clearly concentrated in the west by week 5, although a few hotspot districts are observed in the east. This concentration of ecosyndemic risk remained until weeks 7 and 8. Thereafter, the area of ecosyndemics receded, remains in the west (week 9), and in one district in the east by week 11. 

In January 1998, ecosyndemic risk showed the highest values in the west which diminished as one approached the northeastern section of the subregion. By February, this geographic pattern of ecosyndemic intensity shifted to the southwest coast, and one can observe a greater contrast of ecosyndemic morbidity between the western and eastern sections of the subregion. In March, this distribution remained but within a smaller area of intensity. At the seasonal scale (JFM), ecosyndemic morbidity was concentrated in the west, and one district, San Juan de Bigote. By rank (see Appendix A, Table A5), the districts with the highest and lowest ecosyndemic mordbidity were Rinconada Llicuar (west) and Yamango (east).

#### 3.6.4. Spatial Autocorrelation and Cluster Analysis of Ecosyndemic Index

The global autocorrelation analysis of the ecosyndemic indices indicates generally that ecosyndemic patterns were spatially autocorrelated, except for the first 3 weeks of January and the last week of March (Table 5). The Global Moran’s *I* value ranged from 0.12 (*Z* = 2.06) to 0.64 (*Z* = 9.38). 

**Figure 8:**
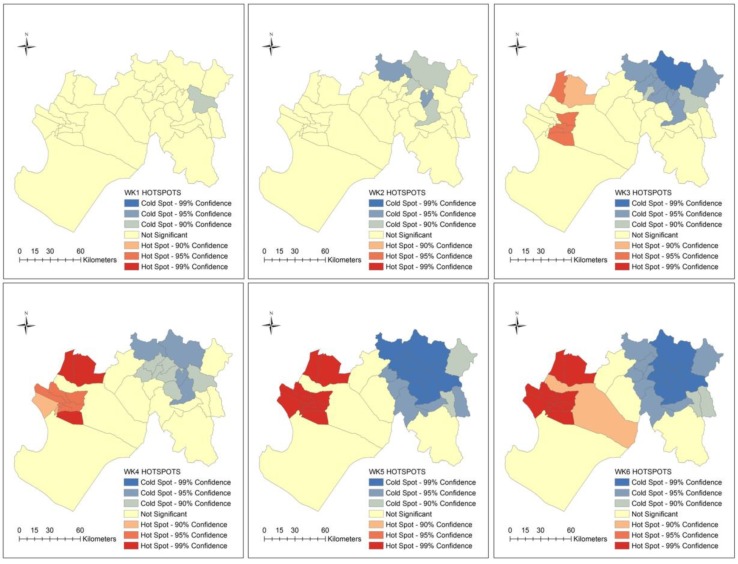

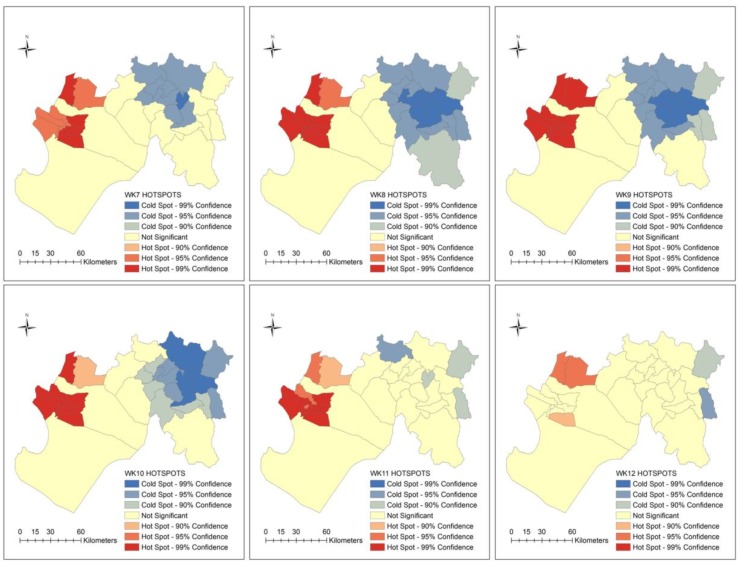
Maps of spatial clusters (hotspots) of the ecosyndemic index at the district-level by week from January to March 1998.

**Figure 9:**
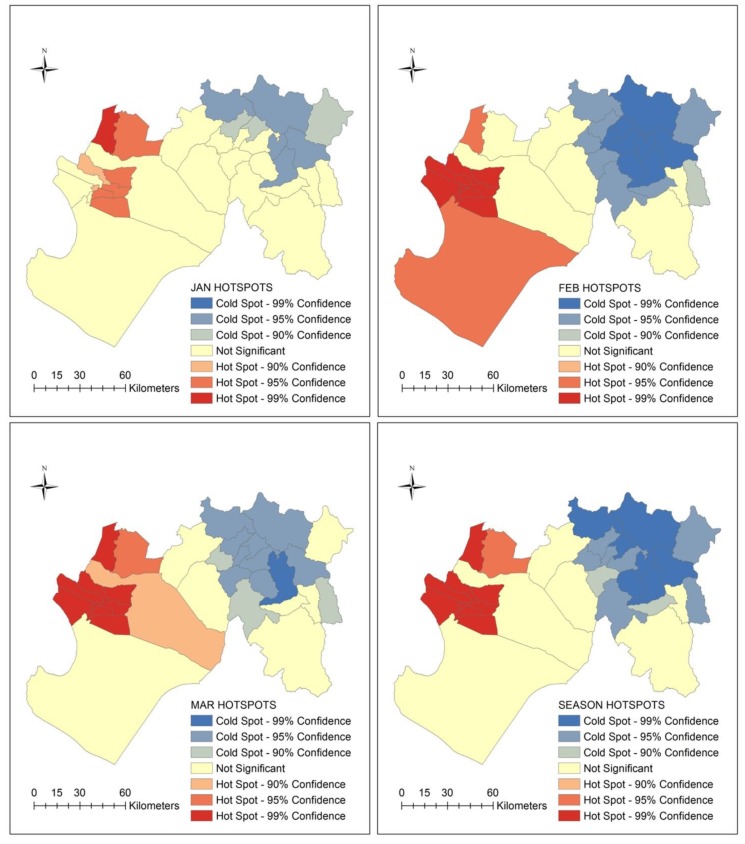
Maps of spatial clusters (hotspots) of the ecosyndemic index at the district-level by month and season from January to March 1998.

The distribution of ecosyndemics clustering is shown at weekly, monthly and seasonal scales in Figure 8 and Figure 9. Using Hotspots analysis (Getis-Ord Gi * statistic), significant spatial clustering (95.0 and 99.0% confidence levels) was identified between weeks 3 and 11. Ecosyndemic hotspots were located in two western cluster areas that bordered the Catacaos district, while coldspots, which fluctuated in spatial coverage, were concentrated in the eastern districts. 

In general, ecosyndemic hotspots were concentrated in 9 to 11 districts for most of the study period (8 weeks). A similar pattern of hotspots is observed at the monthly and seasonal scales, where generally 11 common districts were clustered. The median total incidence rate of these districts (season) was 125.1 per 1000 population. 

#### 3.6.5. Application of Ecosyndemic Index

As proof of concept, two methods were used to assess the usability of the ecosyndemic index. The first method was Pearson’s correlation to understand the potential relationship between ecosyndemics and percent of population that lives in an urban area (URBAN), a proxy variable for disaster impact, such as the number of people affected by El Niño floods (DISASTER), poverty (NBI – basic needs unmet index), without municipal water access (NOMWATER), and without electricity (NOELEC) in the subregion. Among variables, URBAN showed the strongest significant association (*r* = 0.7, *p*-value < 0.01) with weeks 5 and 8, February, and JFM. DISASTER showed moderate associations (*r* = 0.5 to 0.6, *p*-value < 0.01) with Weeks 6, 8, 11 and 12, March, and JFM. NOMWATER and NOELEC were negatively associated (*r* = −0.4 to −0.6, *p*-value < 0.05) with ecosyndemics. NBI was not significantly associated with the ecosyndemic indices. 

**Figure 10:**
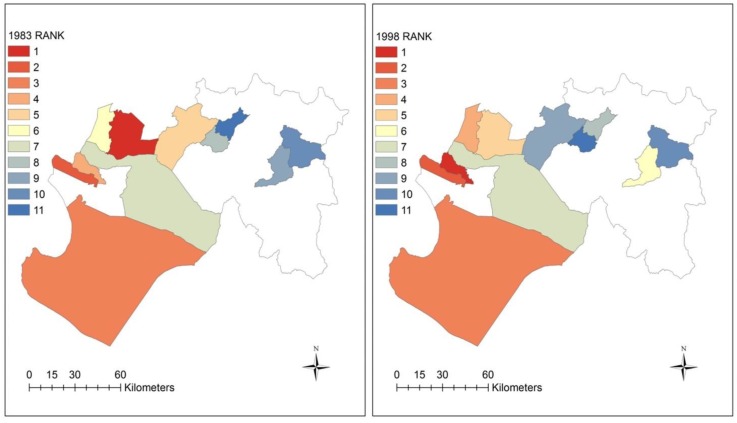
Maps comparing ecosyndemic indices according to rank for 1983 and 1998 across 11 districts in the Piura subregion.

The second method applied the index methodology to another El Niño-related year, 1983. Prior to 1997–1998, the 1982–1983 El Niño was the “El Niño of the 20th century”. It was a mega-El Niño for Peruvians and some may argue it inflicted the most damages because there were no early warnings [52], in part because the buoy system (for monitoring El Niño) had not been implemented across the equatorial Pacific Ocean yet [53]. For this application, select diseases were chosen based on availability of data and commonality between the two El Niño years to generate an index, which was then compared to 1998. Eleven districts were selected for the index, which was based on the incidence of 3 disease variables: EDAS, IRAS, and MALV (see incidence datasets for 1983 and 1998 in Appendix A, Table A6). Figure 10 and Table 5 show maps of the indices for 1983 and 1998 and a comparison by rank. The spatial distribution between the two indices appear to show a similar pattern. As Table 6 indicates, there are 4 districts which have the same ranks in both years. These include La Union, Sechura, Catacaos, and Huancabamba. Pearson’s correlation analysis reveals a positive and strong relationship (*r* = 0.7, *p*-value < 0.01) between the two indices. 

## 4. Discussion

In Piura, numerous disease epidemics were reported during the 1997–1998 El Niño, particularly in the first quarter of 1998 when the ecological effects of several months of anomalous temperatures intersected with torrential rains. In this study, seven climate-sensitive infectious diseases were investigated to characterize the spatial distribution of multi-disease risk using an ecosyndemic approach, and map the potential overlap and clustering of infections at the district-level in Piura. Among these diseases, acute respiratory (non-pneumonia) and diarrheal-related (non-cholera) infections were dominant in terms of their relative contribution (based on %) to morbidity throughout the season (January to March). While both sets of diseases were endemic health problems prior to El Niño variability it is possible that El Nino exacerbated the incidence across the season in Piura and Peru in general. 

Across time there were two temporal patterns among the individual diseases observed from January to March 1998. The first consisted of several diseases (e.g., acute diarrheal disease, pneumonia, and two malarias) with two peaks which occurred between weeks 3 and 6, and weeks 8 and 11. Both spikes in disease coincide with torrential downpours and floods in Piura on January 24 to 25th (~172 mm within 10 h) and approximately during the week of March 8 to 14th. Rainfall estimations are based on a storm analysis using data from the Miraflores meteorological station in the city of Piura (see description of station and data in [1,2]). In general, early March (e.g., week 9) was a significant period for many individual diseases and the total incidence rate (all diseases) for the subregion of Piura.

By district, the spatial distribution of disease burden was highest in the western section and lowest in the eastern section of Piura. In particular, there were nine districts with the highest disease burden, five of which also reported the highest total incidence rates (i.e., Bellavista de la Union, El Tallan, La Arena, La Union, and Rinconada Llicuar). At least two-thirds of these districts were associated with one of the malarias. The flooding in early 1998 led to the breakdown of water and sanitation services [54], as well as an abundance of water which can accumulate in drains, facilitating breeding grounds for the anopheles mosquitoes [55]. The remaining one-third of high burden districts were associated with cholera and conjunctivitis, both diseases associated with hygiene challenges and contaminated water and sanitation infrastructure in developing countries [56,57]. The former disease was clearly linked to El Niño-related temperature changes and flooding. The latter disease was 12 times higher compared to the average disease incidence, and its relationships with climate and El Niño, while known [58], is not well-examined. 

By mapping the spatial distribution of overlapping individual diseases, this study demonstrated that multi-infectious disease risk was highly prevalent across most districts in the subregion. The ecosyndemic mapping results show that geographic patterns of ecosyndemic risk (high) diffused from the central area of the subregion towards a westward concentration in February within a smaller area of intensity. Generally, ecosyndemic risk was highly clustered in two areas that includes 27.3 to 33.3% of districts in the subregion, depending on the temporal scale of analysis. Among high risk ecosyndemic places, five districts were among the top five for total incidence rates and disease burden (ratio): El Tallan, Rinconada Llicuar, Bellavista de la Union, La Arena, and La Union. The latter two districts in particular were identified by Ramírez [1] as climate-impact hotspots in reference to epidemic cholera in 1998. 

The analysis also showed that urbanization (i.e., percent of population living in urban areas) and disaster damages (i.e., number of people affected by floods) were significantly correlated with the ecosyndemic index. The urban association supports evidence of urban vulnerability to hydrometeorological extremes and climatic changes. In part this may be due to the concentration of the susceptible persons exposed to urban pressures such as those contributing to declining infrastructure and sub-standard living conditions [20,59], as well as changing built environments. Interestingly, the basic needs unmet index (NBI) was not significantly correlated with ecosyndemic patterns. Although, 82.0% of districts in Piura reported at least one basic need unmet, social deprivation was widespread prior to 1998. The geography of poverty not only included the high ecosyndemic risk districts in the west mentioned earlier, but also the low ecosyndemic risk districts in the east; thus, a low NBI correlation. This finding may suggest that infrastructure poverty alone is not necessarily an indicator of ecosyndemic risk. More likely, ecological interactions with poverty played a key role for exposures, meaning that multi-disease risk was contingent upon climate, which may have exacerbated social risks and subsequent exposures to multiple pathogens. Furthermore, disease vulnerability generation, particularly in the case of malaria, can also be attributed to human activities, such as land cover change, which may create favorable ecologies for disease vectors [60]. Alternatively, the lack of correlation with infrastructure poverty may also be an issue of scale (e.g., finer unit of analysis with urban areas needed, such as subdistrict) or health indicators (e.g., mortality rather than morbidity). Lastly, the application of the ecosyndemic index to 1983 shows a close resemblance to the pattern of the1998 index; it also showed a strong positive correlation, suggesting the potential utility of the index for public health and disaster risk reduction planning. 

### Study Limitations

Before this approach can be operationalized, a few study limitations must be acknowledged. The first is the challenge of the specificity of the disease data, which may be somewhat imprecise because we utilized suspected and probable (sensitive) counts, as opposed to laboratory-confirmed counts. None-the-less, it is likely that these counts are underestimations because of surveillance challenges. For example, asymptomatic cases can be as high 60.0% to 75.0% for diseases such as malaria and cholera [58,61]. Furthermore, we did not have access to disease and population data stratified by age structure, which may help to account for differential susceptibilities among different groups of people. A second limitation is the ecological fallacy, which assumes that what is observed at one scale cannot be applied to another. Thus, the findings in this study do not assume that all individuals within districts experience the same level of exposure to ecosyndemic risk. A third limitation is that the severity and comprehensiveness of an ecosyndemic is difficult to estimate. While we examined seven critical climate-sensitive diseases, other infections which were reported in Piura were not included such as bartonellosis, leptosporisis, and latigazo (*Paederus irritans*) [30]. This challenge could be addressed, however, by supporting geographic population-level studies with investigations that measure the presence of multiple diseases at the individual-level (e.g., see [62]). Lastly, the time frame was limited to one event; therefore, we cannot generalize the findings without a more comprehensive study that includes more than one event as well as consider the presence of ecosyndemics in non-El Niño years. 

## 5. Conclusions

This study explored and developed an ecosyndemic approach for mapping multi-disease risk in northern Peru during the extreme El Niño in early 1998. Two methodologies were used to investigate the potential simultaneous spatial and temporal overlap of seven climate-sensitive diseases within the geographic subregion of Piura. The first approach presented a practical descriptive method for public health practitioners that tallied the number of diseases present in a district and then classified districts by number of diseases present. This method could be readily implemented using routinely collected surveillance data in Piura and the region. The second mapping approach presented a more complex analysis that utilized data reduction methods (e.g., PCA) to capture an aggregate geographic picture of multi-disease risk that considers interaction between diseases and is represented by a unitless index. To the knowledge of the authors, this is the first study to map and investigate the geographic distribution of ecosyndemics. 

In sum, this study demonstrated that many districts across Piura faced synchronous outbreaks of human diseases over several weeks, which contributed to an excessive burden of disease in two hotspot areas in the subregion. It was also shown that urbanization and disaster impacts were potential correlates of risk, suggesting that urban populations were more susceptible to ecosyndemic morbidity and the impacts of climate-related hazards. Although the index requires further validation, the ecosyndemic measure shows promise for applications to future El Niños and other hydrometeorological extreme events. With growing public health concerns of a changing climate, such information may assist to identify hotspots of multi-disease risk, better target interventions, and improve information for early warning systems in the region. Moreover, it supports efforts for a multi-disease based, interprogrammatic and intersectoral approach to control and prevention, such as the one proposed for neglected tropical diseases in Latin America and the Caribbean by PAHO [34]. Although the weather, water and climate-related hazard context was important for El Niño-related ecosyndemics in 1998, understanding root causes of multi-disease risk will require a more comprehensive analysis of climate and societal determinants and interactions. While climatic changes may influence disease and human ecology, human activities, poverty and other social factors increase population exposure and sensitivity to infectious agents and disasters. Thus, future work should include an analysis that measures the effects of both El Niño (e.g., SST, air temperature and rainfall) and social parameters on ecosyndemics. This should be carried out across several events to characterize the quasi-periodicity of risk across time and space. Furthermore, and importantly, future efforts should include the participation of and collaboration with local public health practitioners to verify and interpret ecosyndemic mapping and recommend actionable applications for regional user needs and priorities.

## Figures and Tables

**Table 1:** Relative contribution (percentage) of each disease to total disease burden (12 weeks, January to March 1998).

	WEEK	EDAS	CHOL	IRAS	PNEU	MALFP	MALV	CONJ
January	1	**30.0**	0.1	50.2	4.6	7.7	6.2	1.1
	2	28.2	0.1	52.2	4.0	7.5	6.3	1.7
	3	28.9	0.2	49.9	4.2	**8.2**	**7.7**	0.9
	4	26.5	1.6	58.4	3.7	5.5	4.0	0.3
February	5	29.8	3.5	53.0	4.6	4.5	4.1	0.5
	6	24.1	4.7	**60.2**	3.6	3.4	3.0	1.1
	7	25.2	6.6	57.0	3.6	3.2	2.8	1.6
	8	27.1	**7.2**	51.2	3.1	3.8	2.9	4.8
March	9	20.2	5.0	55.0	3.6	3.5	3.2	9.6
	10	22.6	4.1	49.8	4.8	4.5	3.7	**10.4**
	11	19.0	3.9	56.6	4.1	4.2	4.7	7.5
	12	18.2	3.8	57.9	**5.7**	5.2	4.8	4.4
SEASON	ALL	**24.6**	3.7	**54.5**	4.1	4.8	4.2	4.0

Highest percent for each disease in bold.

**Table 2:** Disease incidence rate (per 1000 population) by district (season).

District	EDAS	CHOL	IRAS	PNEU	MALFP	MALV	CONJ	TOTAL
Bellavista De La Union	38.2	3.3	128.9	10.9	3.9	18.5	2.4	206.3
Bernal	12.2	4.5	47.4	1.6	1.3	5.8	1.3	74.2
Buenos Aires	15.1	0	67.3	6.7	0	1	6.7	96.8
Canchaque	25.1	1.1	54.5	12.9	1.2	0.4	4.3	99.4
Castilla	28.1	7.2	59.1	2	3.1	0.7	3.5	103.7
Catacaos	21.2	3.7	40.5	7.7	30.6	12.3	3.3	119.2
Chalaco	13.3	1	21.1	1.8	0	0	0.8	37.9
Chulucanas	19.6	2.5	50.9	7	0.5	0.4	1.2	82.1
Cristo Nos Valga	16.1	14.6	27.1	0	0.7	2.5	1.4	62.5
Cura Mori	12	0.9	52.3	7	27.5	25.5	0	125.1
El Carmen de la Frontera	12.7	0.4	12.2	1.9	0	0.2	0.5	28
El Tallan	24.1	1.8	94.4	13.3	12.1	9.2	6.3	161.2
Frias	22.3	0.1	21.3	4.5	0.2	0.3	1.8	50.5
Huancabamba	14.9	0.5	13.5	1.8	0	0.4	0.8	31.9
Huarmaca	26	0.5	5.6	2.8	0.2	1.4	0.9	37.4
La Arena	25.2	2.1	84.2	7.5	16.3	29.2	1.1	165.6
La Matanza	29.8	0.5	33.3	5	0.2	0.2	3	72
La Union	31.2	5.7	75.9	4.4	3.2	22.4	3.9	146.7
Lalaquiz	32.5	0.3	5.6	5.8	1	2.7	1.4	49.3
Morropon	7.5	0.7	23.4	5.7	0.2	0.9	6.9	45.3
Pacaipampa	10	0	5.8	1.3	0	0.1	0.6	17.8
Piupa	25.9	4.9	75.7	2.1	2.3	0.4	5.8	117.1
Rinconada Llicuar	51.8	9.6	33.5	1.2	0.8	12.7	66.1	175.7
Salitral	21.1	2.6	53	3.2	1.2	1.5	8.2	90.6
San Juan de Bigote	36	10.6	58.1	0.9	3.2	1.8	10.5	121.2
San Miguel de el Faique	33.2	0.4	59.7	5.5	1.5	1.9	7.8	110
Santo Catalina Mossa	12.8	3.4	26.2	3.4	0.4	1.3	6.5	54
Santo Domingo	19	0	57.1	5.2	0	0.7	6.2	88.2
Sechura	41.7	9	64.8	2.3	0.1	0.7	5.7	124.3
Sondor	11.4	0	6.7	3.7	0	1.2	0.1	23.1
Sondorillo	16.2	0.7	7	0.7	0	1.9	7.4	33.9
Vice	34.5	2.7	67.1	2.9	0.4	4.6	1.7	113.9
Yamango	3.8	0.4	3.4	0.5	0	0.7	0.7	9.5
Average	22.5	2.9	43.5	4.3	3.4	5	5.4	87.1
Max	51.8	14.6	128.9	13.3	30.6	29.2	66.1	206.3
Min	3.8	0	3.4	0	0	0	0	9.5
Std.	10.9	3.6	30.2	3.4	7.5	8	11.3	50.2

**Table 3:** Disease incidence burden ratio (compared to the average) by district.

District	EDAS	CHOL	IRAS	PNEU	MALFP	MALV	CONJ	TOTAL
Bellavista De La Union	2	1	3	3	1	4	0	2
Bernal	1	2	1	0	0	1	0	1
Buenos Aires	1	0	2	2	0	0	1	1
Canchaque	1	0	1	3	0	0	1	1
Castilla	1	2	1	0	1	0	1	1
Catacaos	1	1	1	2	9	2	1	1
Chalaco	1	0	0	0	0	0	0	0
Chulucanas	1	1	1	2	0	0	0	1
Cristo Nos Valga	1	5	1	0	0	1	0	1
Cura Mori	1	0	1	2	8	5	0	1
El Carmen de la Frontera	1	0	0	0	0	0	0	0
El Tallan	1	1	2	3	4	2	1	2
Frias	1	0	0	1	0	0	0	1
Huancabamba	1	0	0	0	0	0	0	0
Huarmaca	1	0	0	1	0	0	0	0
La Arena	1	1	2	2	5	6	0	2
La Matanza	1	0	1	1	0	0	1	1
La Union	1	2	2	1	1	5	1	2
Lalaquiz	1	0	0	1	0	1	0	1
Morropon	0	0	1	1	0	0	1	1
Pacaipampa	0	0	0	0	0	0	0	0
Piupa	1	2	2	0	1	0	1	1
Rinconada Llicuar	2	3	1	0	0	3	12	2
Salitral	1	1	1	1	0	0	2	1
San Juan de Bigote	2	4	1	0	1	0	2	1
San Miguel de el Faique	1	0	1	1	0	0	1	1
Santo Catalina Mossa	1	1	1	1	0	0	1	1
Santo Domingo	1	0	1	1	0	0	1	1
Sechura	2	3	1	1	0	0	1	1
Sondor	1	0	0	1	0	0	0	0
Sondorillo	1	0	0	0	0	0	1	0
Vice	2	1	2	1	0	1	0	1
Yamango	0	0	0	0	0	0	0	0

**Table 4:** Principal component analysis output for the ecosyndemic index by month.

	Comp.	EDAS	CONJ	IRAS	MALFP	MALV	PNEU	CHOL	Var. %	Cumul. %
January	1	0.34	**0.64**	**0.69**	0.00	0.07	**0.92**	−0.02	34.08	34.08
	2	−0.08	−0.11	0.20	**0.92**	**0.95**	0.10	−0.02	26.52	60.60
	3	**0.78**	0.23	0.45	−0.04	−0.03	−0.12	**0.93**	16.28	76.87
February	1	**0.64**	−0.17	**0.94**	0.08	0.25	**0.73**	0.13	37.48	37.48
	2	**0.67**	**0.78**	0.13	−0.09	0.15	−0.33	**0.82**	27.06	64.54
	3	0.01	0.03	0.14	**0.94**	**0.87**	0.38	−0.01	15.53	80.07
March	1	0.18	−0.02	**0.79**	**0.82**	**0.75**	**0.79**	−0.05	38.76	38.76
	2	**0.88**	**0.87**	0.16	−0.07	0.41	−0.21	**0.69**	29.43	68.19

Factor loadings above 0.60 are highlighted (bold).

**Table 5:** Global index of spatial autocorrelation.

Index	*I*	*Z*	*P*
WK1	−0.06	−0.37	0.71
WK2	0.09	1.65	0.10
WK3	0.09	1.58	0.11
WK4	0.16	2.54	0.01
WK5	0.47	6.75	0.00
WK6	0.50	7.30	0.00
WK7	0.26	3.86	0.00
WK8	0.53	7.46	0.00
WK9	0.64	9.38	0.00
WK10	0.52	7.75	0.00
WK11	0.22	3.55	0.00
WK12	0.00	0.40	0.69
J	0.12	2.06	0.04
F	0.46	6.55	0.00
M	0.49	7.11	0.00
JFM	0.54	7.67	0.00

I: the Global Moran’s *I* coefficient; Z: the Global Moran’s *I* statistic value; *P*: *p*-value for the Global Moran’s *I* statistic.

**Table 6:** Comparison of 1983 and 1998 ecosyndemic indices by rank across 11 districts.

District	1983 Index	Rank	1998 Index	Rank
Castilla	1.00	1	0.57	5
**La Union**	0.95	**2**	0.96	**2**
**Sechura**	0.82	**3**	0.81	**3**
La Arena	0.64	4	1.00	1
Chulucanas	0.47	5	0.37	9
Piura	0.45	6	0.65	4
**Catacaos**	0.30	**7**	0.45	**7**
Morropon	0.21	8	0.00	11
Canchaque	0.18	9	0.48	6
**Huancabamba**	0.06	**10**	0.04	**10**
Santo Domingo	0.00	11	0.41	8

Districts with common ranks are bold.

## Appendix A

**Table A1:** Disease incidence rates (per 1000 population) by week.

	EDAS	CHOL	IRAS	PNEU	MALFP	MALV	CONJ	Total Rate
WK1	1.5	0.0	2.4	0.2	0.4	0.3	0.1	4.9
WK2	1.6	0.0	3.0	0.2	0.4	0.4	0.1	5.8
WK3	2.0	0.0	3.5	0.3	0.6	0.5	0.1	6.9
WK4	2.3	0.1	5.1	0.3	0.5	0.3	0.0	8.7
WK5	2.5	0.3	4.5	0.4	0.4	0.3	0.0	8.5
WK6	2.1	0.4	5.2	0.3	0.3	0.3	0.1	8.6
WK7	2.0	0.5	4.6	0.3	0.3	0.2	0.1	8.1
WK8	2.5	0.7	4.7	0.3	0.3	0.3	0.4	9.1
WK9	2.3	0.6	6.2	0.4	0.4	0.4	1.1	11.4
WK10	1.8	0.3	3.9	0.4	0.4	0.3	0.8	7.9
WK11	1.7	0.3	5.0	0.4	0.4	0.4	0.7	8.8
WK12	1.2	0.2	3.8	0.4	0.3	0.3	0.3	6.5

**Table A2:** Presence of diseases by district (Week 9 example).

District	EDAS	CHOL	IRAS	PNUE	MALFP	MALV	CONJ
**Bellavista de la Union**	X	X	X	X	X	X	—
Bernal	X	X	X	X	X	X	X
Buenos Aires	X	—	X	X	—	—	X
**Canchaque**	X	—	X	X	X	—	X
**Castilla**	X	X	X	X	X	X	X
Catacaos	X	X	X	X	X	X	X
**Chalaco**	X	—	X	X	—	—	—
Chulucanas	X	X	X	X	X	X	X
Cristo Nos Valga	X	X	X	—	—	X	X
Cura Mori	X	X	X	X	X	X	—
El Crmen de la Frontera	X	X	X	X	—	—	X
El Tallan	X	X	X	X	X	X	—
Frias	X	—	X	X	—	—	X
Huancabamba	X	X	X	X	—	—	X
**Huarmaca**	X	X	X	X	—	—	—
La Arena	X	X	X	X	X	X	X
**La Matanza**	X	X	X	X	—	—	X
La Union	X	X	X	X	X	X	X
Lalaquiz	X	—	X	X	—	—	—
Morropon	X	X	X	X	—	X	X
**Pacaipampa**	X	—	X	X	—	—	X
Piura	X	X	X	X	X	X	X
**Rinconada Llicuar**	X	X	X	—	X	X	X
Salitral1	X	X	X	X	X	X	X
San Juan De Bigote	X	X	X	—	—	—	X
San Miguel De El Faique	X	—	X	X	X	—	X
Santa Catalina De Mossa	X	X	X	X	X	—	X
Santo Domingo	X	—	X	X	—	—	X
Sechura	X	X	X	X	X	—	X
Sondor	X	—	X	X	—	X	—
**Sondorillo**	X	—	—	—	—	X	X
Vice	X	X	X	X	X	X	X
Yamango	X	X	X	X	—	—	X

Districts with 7 diseases are highlighted in bold.

**Table A3:** Correlations between disease variables.

	EDAS	CHOL	IRAS	PNEU	MALFP	MALV	CONJ
EDAS	1	0.5 **	0.5 **	0.1	0	0.2	0.5 **
CONJ	0.5 **	0.4 *	0	−0.1	−0.1	0.1	1
IRAS	0.5 **	0.2	1	0.6 **	0.3	0.5 **	0
MALFP	0	0	0.3	0.4 *	1	0.7 **	−0.1
MALV	0.2	0.1	0.5 **	0.4 *	0.7 **	1	0.1
PNEU	0.1	−0.3	0.6 **	1	0.4 *	0.4 *	−0.1
CHOL	0.5 **	1	0.2	−0.3	0	0.1	0.4 *

Statistical significance at the 95.0 % (*) and 99.0 % (**) levels are indicated.

**Table A4:** Principal component analysis output for ecosyndemic indices by week and season.

	Comp.	EDAS	CHOL	IRAS	PNEU	MALFP	MALV	CONJ	Var. %	Cumul. %
WK 1	1	0.14	−0.08	0.07	0.48	0.90	0.86	−0.23	28.32	28.32
	2	0.53	−0.07	0.77	0.14	0.01	−0.01	0.82	23.59	51.92
	3	−0.04	0.89	0.45	0.52	0.05	−0.04	−0.12	15.49	67.41
WK 2	1	0.39	−0.32	0.86	0.85	0.04	0.01	0.73	32.21	32.21
	2	−0.09	−0.06	0.07	0	0.93	0.91	−0.01	25.44	57.65
	3	0.80	0.60	0.13	0.08	0.01	−0.15	−0.32	15.21	72.86
WK 3	1	0.71	−0.07	0.87	0.78	−0.12	0.06	0.03	28.71	28.71
	2	−0.10	0.06	0.06	−0.07	0.94	0.96	−0.11	24.84	53.55
	3	0.39	0.65	−0.03	−0.33	0.03	−0.08	0.75	17.65	71.20
WK4	1	0.84	0.78	0.73	0.35	−0.06	−0.10	0.65	37.52	37.52
	2	0.03	−0.03	0.43	0.61	0.85	0.87	−0.07	26.25	63.77
WK5	1	0.49	0	0.86	0.72	−0.07	0.34	0.79	43.26	43.26
	2	0.03	0.07	0.27	0.46	0.93	0.79	−0.12	20.88	64.13
	3	0.74	0.94	0.25	−0.06	0.10	0.01	0.16	15.24	79.37
WK6	1	0.80	0.83	0.55	0.24	−0.19	0.29	0.10	38.38	38.38
	2	0.24	−0.12	0.30	0.50	0.85	0.75	0.15	19.08	57.46
	3	0.30	−0.15	0.64	0.68	0.21	−0.17	−0.67	14.50	71.96
WK7	1	0.83	0.78	0.64	0.05	−0.02	0.04	0.60	29.44	29.44
	2	0.17	−0.10	0.16	0.07	0.90	0.94	−0.33	28.26	57.70
	3	0.03	−0.42	0.36	0.96	0.19	−0.04	0.09	16.66	74.36
WK8	1	0.93	0.82	0.44	−0.11	−0.08	−0.02	0.75	34.22	34.22
	2	0.13	−0.05	0.64	0.69	0.79	0.86	−0.16	31.60	65.82
WK9	1	0.94	0.71	0.15	−0.30	0.15	0.84	0.93	45.02	45.02
	2	0.14	−0.13	0.80	0.79	0.72	0.38	−0.08	27.16	72.18
WK10	1	0.77	0.85	0.25	0.11	−0.28	0.31	0.86	40.55	40.55
	2	0.35	0.13	0.69	0.43	0.83	0.81	−0.01	23.06	63.61
WK11	1	0.07	−0.08	0.75	0.69	0.81	0.81	−0.02	33.74	33.74
	2	0.92	0.90	0.17	−0.08	−0.09	−0.02	0.60	29.42	63.16
WK12	1	−0.29	0.06	0.75	0.79	0.90	0.76	−0.08	38.40	38.40
	2	0.31	0.86	0.52	−0.03	−0.04	−0.18	0.90	27.82	66.23
SEASON	1	0.25	−0.06	0.74	0.78	0.76	0.81	−0.09	36.72	36.72
	2	0.82	0.78	0.35	−0.21	−0.13	0.18	0.76	28.18	64.89

**Table A5:** Ecosyndemic index and rank by district for the season (JFM) in 1998.

DISTRICT	JFM INDEX	RANK
Rinconada Llicuar	1	1
Bellavista De La Union	0.9	2
La Arena	0.78	3
La Union	0.72	4
El Tallan	0.69	5
Catacaos	0.62	6
San Juan De Bigote	0.61	7
Sechura	0.61	8
Cura Mori	0.61	9
Vice	0.49	10
Castilla	0.47	11
Piura	0.46	12
San Miguel De El Faique	0.46	13
Canchaque	0.44	14
Cristo Nos Valga	0.39	15
Salitral1	0.36	16
Chulucanas	0.34	17
Buenos Aires	0.33	18
La Matanza	0.32	19
Santo Domingo	0.31	20
Bernal	0.3	21
Lalaquiz	0.3	22
Santa Catalina De Mossa	0.23	23
Frias	0.22	24
Huarmaca	0.2	25
Morropon	0.17	26
Sondorillo	0.15	27
Chalaco	0.13	28
Huancabamba	0.12	29
El Carmen De La Frontera	0.1	30
Sondor	0.09	31
Pacaipampa	0.05	32
Yamango	0	33

**Table A6:** Disease incidence data (per 1000 population) for ecosyndemic index construction and comparison between El Niño years, 1983 and 1998.

	1983			1998		
District	EDAS	IRAS	MALV	EDAS	IRAS	MALV
Canchaque	23.6	1.8	4.6	25.1	54.5	0.4
Castilla	51.4	52.4	24.1	28.1	59.1	0.7
Catacaos	23.2	6.9	20.8	21.2	40.5	12.3
Chulucanas	20.7	38.1	0.9	19.6	50.9	0.4
Huancabamba	5.9	9.3	0.3	14.9	13.5	0.4
La Arena	30.0	15.5	58.9	25.2	84.2	29.2
La Union	80.7	12.1	35.9	31.2	75.9	22.4
Morropon	17.1	11.8	4.0	7.5	23.4	0.9
Piura	30.7	24.6	3.6	25.9	75.7	0.4
Santo Domingo	1.7	6.4	0.3	19.0	57.1	0.7
Sechura	41.9	55.3	0.7	41.7	64.8	0.7

EDAS: acute diarrheal diseases that do not include cholera; IRAS: acute respiratory diseases that do not include pneumonia; and MALV: malaria *P. vivax.*

## References

[1] Ramírez I.J. (2015). Cholera resurgence in Piura, Peru: Examining climate associations during the 1997–98 El Niño. GeoJournal.

[2] Ramírez I.J., Grady S.C. (2016). El Niño, climate and cholera associations in Piura, Peru, 1991–2001: A wavelet analysis. EcoHealth.

[3] Ramírez I.J., Briones F. (2017). Understanding the El Niño costero of 2017: The definition problem and challenges of climate forecasting and disaster responses. Int. J. Disaster Risk Sci..

[4] Glantz M.H., Naranjo L., Baudoin M., Ramírez I.J. (2018). What does it mean to be El Niño Ready?. Atmosphere.

[5] Naranjo L., Glantz M.H., Temirbekov S., Ramírez I.J. (2018). El Niño and the Köppen-Geiger classification: A prototype concept and methodology for mapping impacts in Central America and the Circum-Caribbean. Int. J. Disaster Risk Sci..

[6] Glantz M.H. (2015). Shades of chaos: Lessons learned about lessons learned about forecasting El Niño and its impacts. Int. J. Disaster Risk Sci..

[7] El Niño and Health: A Global Overview—January 2016. http://www.who.int/hac/crises/el-Niño/who_el_Niño_and_health_global_report_21jan2016.pdf?ua=1.

[8] Ramírez I.J., Grady S.C., Glantz M.H. (2013). Reexamining El Niño and cholera in Peru: A climate affairs approach. Weather Clim. Soc..

[9] Gagnon A.S., Smoyer-Tomic K.E., Bush A.B. (2002). The El Niño southern oscillation and malaria epidemics in South America. Int. J. Biometeorol..

[10] Stewart-Ibarra A., Lowe R. (2013). Climate and non-climate drivers of dengue epidemics in Southern Coastal Ecuador. Am. J. Trop. Hyg. Med..

[11] Ruiz E.F., Vasquez-Galindo C.M., Aquije-Pariona X.M., Smith Torres-Roman J. (2018). Outbreaks caused by Aedes aegyptis due to El Niño in a coastal area of Peru. Travel Med. Infect. Dis..

[12] Bertuzzo E., Azaele S., Maritan A., Gatto M., Rodriguez-Iturbe I., Rinaldo A. (2008). On the space-time evolution of a cholera epidemic. Water Res. Res..

[13] Mari L., Bertuzzo E., Righetto L., Cassagrandi R., Gatto M., Rodriguez-Iturbe I., Rinaldo A. (2012). Modelling cholera epidemics: The role of waterways, human mobility and sanitation. J. R. Soc. Interface.

[14] Cutter S.L., Solecki W., Bragado N., Carmin J., Fragkias M., Ruth M., Wilbanks T.J., Melillo J.M., Richmond T., Yohe G.W. (2014). Urban systems, infrastructure, and vulnerability. Climate Change Impacts in the United States: The Third National Climate Assessment.

[15] Pan American Health Organization (PAHO) El Nino-Southern Oscillation and Health. http://www.who.int/globalchange/publications/factsheets/el-nino-and-health/en/.

[16] Vargas C.M., Cupe J.A. Desastres Naturales Y Prevencion De Enfermedades [Natural Disasters and Prevention of Communicable Diseases]. http://www.scielo.org.pe/scielo.php?script=sci_arttext&pid=S1728-59172017000100001.

[17] Thomson M.C., Mason S.J., Phindela T., Connor S. (2005). Use of rainfall and sea surface temperature monitoring for malaria warning in Botswana. Am. J. Trop. Hyg. Med..

[18] Ceccato P., Connor S.J., Jeanne I., Thomson M.C. (2005). Application of geographic information systems and remote sensing technologies for assessing and monitoring malaria risk. Parassitologia.

[19] Mantilla G., Oliveros H., Barnston A.G. (2009). The role of ENSO in understanding changes in Colombia’s annual malaria burden by region, 1960–2006. Malar. J..

[20] Kjellstrom T., Friel S., Dixon J., Corvalan C., Rehfuess E., Campbell-Lendrum D., Gore F., Bartram J. (2007). Urban environmental health hazards and health equity. J. Urban Health.

[21] Gamble J.L., Balbus J., Berger M., Bouye K., Campbell V., Chief K., Conlon K., Crimmins A.R., Flanagan B., Gonzalez-Maddux C.M., Crimmins A., Balbus J., Gamble J.L., Beard C.B., Bell J.E., Dodgen D., Eisen R.J., Fann N., Hawkins M.D., Herring S.C. (2016). Ch.9: Populations of concern. The Impacts of Climate Change on Human Health in the United States: A Scientific Assessment.

[22] Utzinger J., Keiser J. (2006). Urbanization and tropical health—Then and now. Ann. Trop. Med. Parasitol..

[23] Adelekan I.O. (2010). Vulnerability of poor urban coastal communities to flooding in Lagos, Nigeria. Environ. Urban.

[24] Tong M.X., Hansen A., Hanson-Easey S., Cameron S., Xiang J., Liu Q., Sun Y., Weinstein P., Han G.S., Williams C. (2015). Infectious diseases, urbanization and climate change: Challenges in future China. Int. J. Environ. Res. Public Health.

[25] Singer M. (2009). Introduction to Syndemics: A Critical Systems Approach to Public and Community Health.

[26] Singer M., Bulled N., Ostrach B., Mendenhall E. (2017). Syndemics and the biosocial conception of health. Lancet.

[27] Singer M. (2013). Respiratory health and ecosyndemics in a time of global warming. Health Soc. Rev..

[28] Gueri A. Lessons Learned: Health Effects of El Niño in Peru. http://helid.digicollection.org/en/d/Jdi019e/2.html.

[29] Sandoval P.S. (1999). Evaluación De Daños Y Acciones Del Fenómeno El Niño (Evaluation of Damages Caused by the El Niño Phenomenon and Actions Taken).

[30] Ministerio de Salud en Piura [Ministry of Health] (MINSA) (2001). Enfermedades Emergentes—Reemergentes Y Modificaciones en El Perfil Epidemiologico Del Noroeste Peruano Post Fenomeno El Niño. [Emerging and Reemerging Diseases and Impacts on Epidemiology in Northern Peru, Post-El Niño].

[31] Lluvia e Inundaciones en Peru Reporte De Situation [Rains and Floods in Peru. Situation Report]. https://reliefweb.int/sites/reliefweb.int/files/resources/Informe_Situacion_13-2017_Peru_Inundaciones_23_abril%5B1%5D.pdf.

[32] Cromley E.K., McLafferty S.L. (2003). GIS and Public Health.

[33] Mclafferty S. (2015). Disease cluster detection methods: Recent developments and public health implications. Ann. GIS.

[34] Ehrenberg J.P., Ault S.K. (2005). Neglected diseases of neglected populations: Thinking to reshape the determinants of health in Latin America and the Caribbean. BMC Public Health.

[35] Hotez P.J., Bottazzi M.E., Franco-Paredes C., Ault S.K., Periago M.R. (2008). The neglected tropical diseases of Latin America and the Caribbean: A review of disease burden and distribution and a roadmap for control and elimination. PLOS Neglected Trop. Dis..

[36] Schneider M.C., Aguilera X.P., da Silva Junior J.B., Ault S.K., Najera P., Martinez J., Requejo R., Nichols R.S., Yadon Z., Silva J.C. (2011). Elimination of neglected diseases in Latin America and the Caribbean: A mapping of selected diseases. PLOS Neglected Trop. Dis..

[37] United Nations Development Programme (UNDP) Human Development Index. http://hdr.undp.org/en/content/human-development-index-hdi.

[38] Cutter S.L., Boruff B.J., Shirley W.L. (2003). Social vulnerability to environmental hazards. Soc. Sci. Q..

[39] Instituto Nacional de Estadística e Informática (National Institute for Statistics and Information, Peru) (INEI) Necesidades Basicas Insatisfechas (Basic Needs Unmet). http://proyectos.inei.gob.pe/web/biblioineipub/bancopub/Est/Lib0068/n00.htm.

[40] Organization for Economic Co-operation and Development (OECD) Handbook for constructing composite indicators: methodology and user guide. https://www.oecd.org/sdd/42495745.pdf.

[41] Tate E. (2012). Social vulnerability indices: A comparative assessment using uncertainty and sensitivity analysis. Nat. Hazards.

[42] Mazziota M., Pareto A. (2013). Methods for constructing composite indices: One for all or all for one?. Riv. Ital. Econ. Demogr. Stat..

[43] Confalonieri U.E.C., Marinho D.P., Rodriguez R.E. (2009). Public health vulnerability to climate change in Brazil. Clim. Res..

[44] Rodriguez R., Macabres A., Luckman B., Evans M., Masiokas M., Ektvedt T.M. (2005). “El Niño” events recorded in dry-forest species of the lowlands of northwest Peru. Dendrochronologia.

[45] INEI (2000). Encuesta Demográfica y de Salud Familiar 2000 (Study of Family Demography and Health 2000).

[46] Monthly Atmospheric and SST Indices. http://www.cpc.ncep.noaa.gov/data/indices/.

[47] INEI (1995). Impacto Del Fenomeno Del Niño Durante 1982/83 en El Departamento De Piura (Impact from the 1982–83 El Niño in the Department of Piura).

[48] INEI Censo Nacionales 1993 (National Census 1993). http://censos.inei.gob.pe/bcoCuadros/bancocuadro.asp?p=14.

[49] (1998). Fenomeno Del El Niño 1998: Empradonamiento De La Poblacion Residents en Areas Afectadas Por El Fenomeno De El Niño (The 1998 El Niño Phenomenon: Survey of the Population in Areas Affected by the 1998 El Niño Phenomenon).

[50] How Inverse Distance Weighted (IDW) Interpolation Works. http://webhelp.esri.com/arcgisdesktop/9.2/index.cfm?TopicName=How_Inverse_Distance_Weighted_(IDW)_interpolation_works.

[51] Spatial Autocorrelation (Global Moran’s I). http://desktop.arcgis.com/en/arcmap/10.3/tools/spatial-statistics-toolbox/spatial-autocorrelation.htm.

[52] Reports to the Nation: El Niño and Climate Prediction. https://atmos.washington.edu/gcg/RTN/rtnt.html.

[53] History of the Array. http://tao.ndbc.noaa.gov/proj_overview/taohis_ndbc.shtml.

[54] PAHO (1998). Perú: Fenómeno “El Niño”. Informe Estratégico #2.

[55] Rosas-Aguirre A., Llanos-Cuentas A., Speybroeck N., Cook J., Contreras-Mancilla J., Soto V., Gamboa D., Pozo E., Ponce O.J., Pereira M.O. (2013). Assessing malaria transmission in a low endemicity area of North-Western Peru. Malar. J..

[56] Cholera. http://www.who.int/news-room/fact-sheets/detail/cholera.

[57] Alerta Epidemiologica: Conjuntivitis Hermorragico Aguda [Epidemiological Alert: Acute Hemorrhagic Conjunctivitis]. https://www.paho.org/hq/dmdocuments/2013/alertas-epi-2009-9-octubre-conjunctivitis.pdf.

[58] Vamos a Prevenir: Plan De Communicacion: Fen 2015 (Let’s Prevent: Communication Plan for El Niño 2015). http://www.minsa.gob.pe/portada/especiales/2015/fenomeno/index.asp?op=3.

[59] Friel S., Hancock T., Kjellstrom T., McGranahan G., Monge P., Roy J. (2011). Urban health inequities and the added pressure of climate change: An action-oriented research agenda. J. Urban Health.

[60] Vittor A.Y. (2009). Linking deforestation to malaria in the Amazon: characterization of the breeding habitat of the principal malaria vector *Anopheles darling*. Am. J. Trop. Hyg. Med..

[61] Hassanpour G., Mohebali M., Zeraati H., Raeisi A., Keshavarz H. (2017). Asymptomatic malaria and its challenges in the malaria elimination program in Iran: A systematic review. J. Arthropod-Borne Dis..

[62] Carrillo-Hernández M.Y., Ruiz-Saenz J., Jaimes Villamizar L., Gomez-Rangel S.Y., Martinez-Gutierrez M. (2018). Co-circulation and simultaneous co-infection of dengue, chikungunya, and zika viruses in patients with febrile syndrome at the Colombian-Venezuelan border. BMC Infect. Dis..

